# Histone deacetylase inhibitors sodium butyrate and valproic acid delay spontaneous cell death in purified rat retinal ganglion cells

**Published:** 2011-02-05

**Authors:** Julia Biermann, Jennifer Boyle, Amelie Pielen, Wolf Alexander Lagrèze

**Affiliations:** University Eye Hospital Freiburg, Freiburg im Breisgau, Germany

## Abstract

**Purpose:**

Histone deacetylase inhibitors (HDACi) have neuroprotective effects under various neurodegenerative conditions, e.g., after optic nerve crush (ONC). HDACi-mediated protection of central neurons by increased histone acetylation has not previously been demonstrated in rat retinal ganglion cells (RGCs), although epigenetic changes were shown to be associated with cell death after ONC. We investigated whether HDACi can delay spontaneous cell death in purified rat RGCs and analyzed concomitant histone acetylation levels.

**Methods:**

RGCs were purified from newborn (postnatal day [P] 0–P2) rat retinas by immunopanning with antibodies against Thy-1.1 and culturing in serum-free medium for 2 days. RGCs were treated with HDACi, each at several different concentrations: 0.1–10 mM sodium butyrate (SB), 0.1–2 mM valproic acid (VPA), or 0.5–10 nM trichostatin A (TSA). Negative controls were incubated in media alone, while positive controls were incubated in 0.05–0.4 IU/µl erythropoietin. Survival was quantified by counting viable cells using phase-contrast microscopy. The expression of acetylated histone proteins (AcH) 3 and 4 was analyzed in RGCs by immunohistochemistry.

**Results:**

SB and VPA enhanced RGC survival in culture, with both showing a maximum effect at 0.1 mM (increase in survival to 188% and 163%, respectively). Their neuroprotective effect was comparable to that of erythropoietin at 0.05 IU/µl. TSA 0.5–1.0 nM showed no effect on RGC survival, and concentrations ≥5 nM increased RGC death. AcH3 and AcH4 levels were only significantly increased in RGCs treated with 0.1 mM SB. VPA 0.1 mM produced only a slight effect on histone acetylation.

**Conclusions:**

Millimolar concentrations of SB and VPA delayed spontaneous cell death in purified RGCs; however, significantly increased histone acetylation levels were only detectable in RGCs after SB treatment. As the potent HDACi TSA was not neuroprotective, mechanisms other than histone acetylation may be the basis on which SB and VPA are acting in this model. Additional studies are necessary to identify HDACi-targeted genes and pathways involved in RGC protection.

## Introduction

Transcription in eukaryotes is a highly regulated process, and acetylation is now known to play a major role in its epigenetic modification [1]. The acetylation or deacetylation of histone N-terminal tails alters the interaction between histones and DNA in chromatin, and this chromatin remodeling has been identified as a key step in the regulation of gene expression [2,3]. In general, hyperacetylation is associated with transcriptional activation, whereas hypoacetylation is associated with repression. Histone acetyltransferases (HAT) and histone deacetylases (HDAC) represent two enzyme classes that balance the acetylation status in neurons. This acetylation homeostasis is impaired toward deacetylation in neurodegenerative diseases, such as amyotrophic lateral sclerosis, Alzheimer disease, and Parkinson disease [4,5].

Drugs that reduce histone deacetylation in diseased neurons may restore transcriptional balance and hence delay or prevent cell degeneration. Such compounds, known as histone deacetylase inhibitors (HDACi), affect histones as well as transcription factors that are regulated by acetylation [6]. HDACi are divided into four groups: 1. short-chain fatty acids (e.g., sodium butyrate [SB], valproic acid [VPA]), 2. hydroxamic acids (e.g., trichostatin A [TSA], suberoylanilide hydroxamic acid [SAHA]), 3. cyclic tetrapeptides, and 4. benzamides [1]. SB, first synthesized in 1949, and VPA, an anti-epileptic drug, were the first known HDACi and, together with TSA, recently attracted attention as potentially neuroprotective drugs [7]. It has been suggested that their action is linked to a large extent to direct inhibition of HDAC [8], causing histone hyperacetylation. However, the underlying molecular mechanisms are still not fully understood.

In in vitro experiments HDACi protected neurons from glutamate-induced excitotoxicity [9], oxygen-glucose deprivation injury [10], and oxidative stress [11]. They also prolonged the life span of cultured cortical neurons [12] and promoted neuronal growth [13,14]. Furthermore, in vivo investigations demonstrated that HDACi protected neurons exposed to intracerebral hemorrhage [15], ischemic stroke [16], and in chronic neurodegenerative diseases [17-19]. Apart from HDAC inhibition, this neuroprotective effect probably involves multiple other mechanisms of action, including modulation of the extracellular signal-regulated kinase pathway [13] and the inhibition of pro-apoptotic molecules [16] or microglia-mediated inflammation [20].

Several ophthalmologic diseases (e.g., experimental glaucoma, acute optic nerve damage) lead to retinal ganglion cell (RGC) death after changes in transcription of several genes [21,22]. The involvement of histone deacetylation in this pathology has recently been shown by Pelzel et al. who reported a histone H4 deacetylation early after optic nerve crush (ONC) in mice [23]. Yet there are few reports on the effects of HDACi on RGCs. Schwechter et al. demonstrated that TSA caused significant differentiation and neuritogenesis of RGC-5 cells [24]. Recently, we detected a neuroprotective effect of VPA on RGCs after ONC [25] but were unable to verify changes in histone acetylation levels using western blots. However, the literature clearly indicates HDAC involvement in VPA-mediated activity. An interpretation for this discrepancy is that the VPA-induced increase in histone acetylation in RGCs was too small to be detected in full retinal protein extracts because only the RGCs were damaged by ONC, possibly resulting in a rather small signal-to-noise ratio [25].

To overcome this problem, we therefore used purified postnatal rat RGC cultures to quantify and pharmacologically characterize the survival-promoting effect of SB, VPA, and TSA in the present study. In addition we used immunohistochemistry to quantify the expression of acetylated histone proteins (AcH) 3 and 4 in RGCs as markers of HDACi-mediated hyperacetylation. The cell-death model used in this experiment is based on apoptosis induced by neurotrophic deprivation. It results from axotomy before RGC harvesting.

## Methods

### Cell culture

All animals were treated in accordance with the Institute for Laboratory Animal Research (Guide for the Care and Use of Laboratory Animals), and all procedures were approved by the Committee of Animal Care of the University of Freiburg. RGCs were purified by a modification [26] of immunopanning with antibodies against Thy-1.1 specific for RGCs and were cultured in serum-free medium, as previously described [27,28]. Cell culture reagents were obtained from Gibco Invitrogen Karlsruhe, Germany. In brief, rat (Charles River, Sulzfeld, Germany) retinas were extracted from newborn (postnatal day [P]0–P2) Sprague Dawley rats (three pups per six retinas per attempt) and incubated at 37 °C for 20 min in 0.125% trypsin in Ca^2+^/Mg^2+^-free Hank’s balanced salt solution. Enzyme treatment was stopped by washing the tissue twice with Dulbecco’s modified eagle medium (DMEM+GlutaMAX; Gibco Invitrogen) containing 10% horse serum, 10 mM Hepes, 100 units/ml penicillin G (sodium salt) and 100 μg/ml streptomycin sulfate, followed by centrifugation at 140 g for 2 min. To obtain a suspension of single cells, the retinal tissue was triturated with a flame-narrowed glass pipette in 5 ml DMEM containing 10% horse serum.

Prior to this, panning dishes (Falcon, Becton & Dickinson, Heidelberg, Germany) were incubated with goat antimouse immunoglobulin (IgG) antibodies (2 μg/ml; Sigma, Munich, Germany) in Tris-HCl buffer (pH 9.5) for 12 h at 4 °C. The dishes were then washed three times with Dulbecco's phosphate-buffered saline (D-PBS) without magnesium or calcium (Cat. No. 14190–094, Gibco; D-PBS contains potassium chloride 2.67 mM, potassium phosphate monobasic 1.47 mM, sodium chloride 137.93 mM and sodium phosphate dibasic 8.06 mM) followed by incubation with anti-Thy-1.1 (0.8 μg/ml, mouse antirat CD90; Serotec, Düsseldorf, Germany) for at least 2 h at 4 °C in PBS. After removal of the supernatant, the retinal cell suspension was transferred to the panning dish and incubated for 20 min at 37 °C. Dishes were gently swirled every 5 min to ensure contact of all RGCs with the surface of the plate. To remove nonadherent cells, dishes were washed repeatedly with D-PBS and swirled moderately until only adherent cells remained. Washing was monitored under a microscope.

RGCs were mechanically removed from the panning dishes in serum-free DMEM by using a cell scraper. After centrifugation at 140 g for 5 min, purified RGCs were suspended in fresh culture medium and their density was determined by counting an aliquot in a hemocytometer. The cells were seeded in 96-well plates (Greiner Bio-One, Frickenhausen, Germany) at a density of 3,000 cells per well and incubated for 48 h at 37 °C in a humidified atmosphere containing 5% CO_2_. Plates had been previously coated with poly-D-lysine (0.1 mg/ml) followed by laminin (7.5 μg/ml) in DMEM.

### Histone deacetylase inhibitor treatment

Various concentrations of HDACi (all from Sigma) were added to culture medium in quadruplicates to hexaplicates per attempt: SB 0.1, 0.5, 1, 5, 10 mM; VPA 0.1, 0.5, 0.75, 1, 2 mM; and TSA 0.5, 1, 5, 10 nM. SB and VPA were dissolved in sterile water and diluted with pure culture medium. Water-insoluble TSA was dissolved in sterile DMSO (2 mg/ml), and further diluted in methanol and medium. Negative controls of the SB or VPA experiments were cultured in pure media. The TSA control groups received the same amount of vehicle (DMSO and methanol) without TSA. Positive controls were treated with erythropoietin (EPO, Epoetin alfa; Janssen-Cilag GmbH, Neuss, Germany) at concentrations of 0.05 IU/µl, 0.1 IU/µl, 0.2 IU/µl, and 0.4 IU/µl, as the neuroprotective potential of EPO has been extensively evaluated in cultures of RGCs and animal models of optic nerve diseases [29-31].

### Quantification of viable retinal ganglion cells

After a culture period of 2 days, RGC cultures were fixed in 1% glutaraldehyde, rinsed with water, and examined unblinded by phase-contrast microscopy. The number of surviving RGCs was assessed by whole-well counts. Viable ganglion cells were morphologically identified by their phase-bright appearance, intact cell bodies with smooth membranes, and neuritic processes.

### Immunohistochemistry

For immunohistochemistry, a single RGC suspension was prepared as described above. Instead of seeding the cells in 96-well plates, cells were placed in equal parts into petriperm culture dishes (Greiner Bio-One GmbH). These dishes have a clear gas-permeable base membrane. After culturing with or without 0.1 mM SB or 0.1 mM VPA over 24 h, cells on the membrane were fixed using ice-cold methanol (−20 °C) for 10 min. Immunohistochemistry was performed after nonspecific binding was blocked with 10% fetal calf serum for 30 min. After washing with D-PBS, cells were incubated with anti-Thy-1.1 (dilution 1:50, Serotec) overnight at 4 °C to identify RGCs, as it was not possible to avoid minor contamination of the cell suspension with other retinal cells. Thy-1.1 was then conjugated with the corresponding rhodamin secondary antibody (red fluorescence, dilution 1:50; KPL, Gaithersburg, MD). Thereafter, the protocol was repeated, and anti-AcH3 (dilution 1:600; Cell Signaling, Danvers, MA) or anti-AcH4 (dilution 1:200; Cell Signaling) was used as a second primary antibody to investigate the acetylation level in RGCs, with or without HDACi treatment. AcH3 or AcH4 was then conjugated with Cy2™ secondary antibody (green fluorescence, dilution 1:80; Jackson ImmunoResearch, West Grove, PA). Subsequently, the base membranes of the petriperm dishes were cut and covered with embedding medium (Mowiol; Calbiochem, San Diego, CA).

### Acetylation densitometry

Cells were photographed under a fluorescence microscope (Axiophot; Carl Zeiss, Jena, Germany) in a linear modus, using the software of Carl Zeiss Axio vision (Axio Rel. 4.7; Carl Zeiss). The same exposure time was used for all photographs per experiment. The expression pattern of AcH3 and AcH4 in RGCs was compared with and without SB or VPA treatment after 24 h in culture using ImageJ software (Bethesda, MD; open-label analysis). AcH3- or AcH4-positive RGCs were localized through their additional Thy-1.1 immunoreactivity. Single AcH3/4-positive RGCs were encircled with the ImageJ elliptical selection tool. After calibration of the cell-signal threshold by determining background staining, the number of pixels per gray tone was counted and totaled for each cell (n=3 experiments, 60 cells per group) to determine an intensity value per cell. The mean intensity value of each group of SB/VPA-treated RGCs was divided by the mean intensity value of the corresponding control RGCs (pure media). A quotient of >1 represents a hyperacetylation in SB/VPA-treated RGCs; a quotient of 1 means that AcH3/AcH4 was equally expressed in both groups; and a quotient of <1 represents a hypoacetylation in RGCs after treatment.

### Statistical analysis

All averages are presented as means with their corresponding standard error of the mean (SEM). Statistical significance was assessed using the unpaired *t* test (immunohistochemistry) or ANOVA, followed by Tukey–Kramer post hoc testing for multiple comparison procedures (cell-culture experiments). GraphPad InStat and GraphPad Prism software (La Jolla, CA) were used. Differences were considered significant at p<0.05.

## Results

### Cell culture and identification of retinal ganglion cells

**Figure 1:**
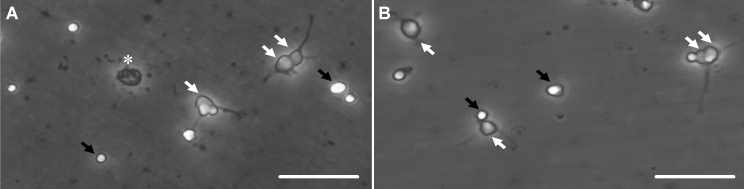
Viable purified retinal ganglion cells in culture after 48 h. **A**, **B**: Representative phase contrast micrographs showing retinal ganglion cell (RGC) controls (**A**, pure media) and RGCs after treatment with histone deacetylase inhibitors (HDACi; **B**, VPA) after 48 h in culture. RGCs (white arrows) can be discriminated from other retinal cells (black arrows) by their large cell diameter and fine neurites with branching growth cones, thus fulfilling the criteria for being counted as viable RGCs. Larger numbers of degenerated RGCs (asterisk in **A**) were present in the control wells. Each scale bar is 50 µm.

The numbers of viable RGCs per well were counted with or without drug treatment after 48 h in culture, using phase-contrast microscopy. RGCs can be discriminated from other retinal cells by their large cell diameter and fine neurites with branching growth cones (Figure 1, white arrows). Degenerated apoptotic RGCs (see asterisk in Figure 1A) were not counted. Despite extensive washing of the panning plate after binding of RGCs to the Thy-1.1-coated plate, a small number of other retinal cells (Figure 1, black arrows) were still present in culture in each group.

### Histone deacetylase inhibitor-induced neuroprotection of retinal ganglion cells

**Figure 2:**
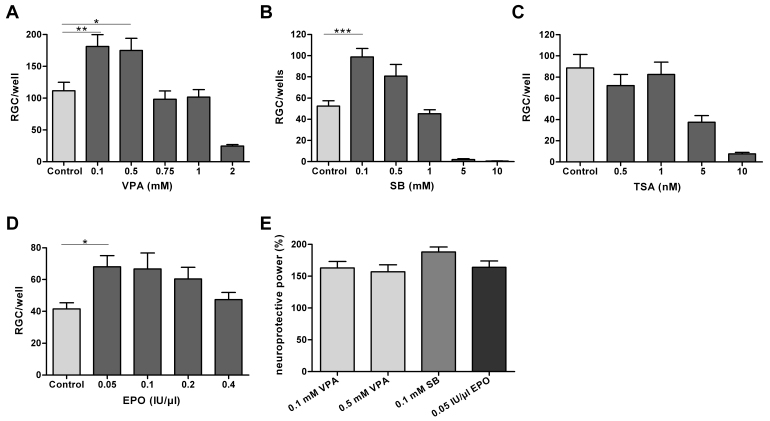
Retinal ganglion cell survival. **A**-**D**: Graphs showing the dose-dependent effect of histone deacetylase inhibitors and erythropoietin on retinal ganglion cell (RGC) survival after 48 h in culture. Data are presented as means and standard error of the mean (each diagram summarizes four to six culture experiments; each of the experiments tested the drug in quadruplicates to hexaplicates of indicated concentrations). **A**: Compared with untreated controls, valproic acid (VPA) 0.1 and 0.5 mM significantly increased RGC survival to 163% (**p<0.01) and 157% (*p<0.05), respectively. Higher VPA concentrations (0.75 and 1.0 mM) had no protective effect, while VPA 2 mM significantly decreased RGC survival to 22% (p<0.001). **B**: Sodium butyrate (SB) 0.1 mM significantly increased RGC survival to 188% (***p<0.001), while a nonsignificant protective effect was seen with SB 0.5 mM. SB 1.0 mM did not affect RGC survival compared to controls. Higher concentrations of SB (≥5 mM) significantly decreased RGC survival. **C**: Trichostatin A (TSA) 0.5 and 1.0 nM had no effect on RGC survival, while TSA 5 nM and 10 nM significantly decreased RGC survival to 40% (p<0.01) and 8% (p<0.001), respectively. **D**: Erythropoietin (EPO) 0.05 IU/µl increased RGC survival to 164% (*p<0.05), while higher concentrations of EPO were less effective. **E**: Histone deacetylase inhibitors (HDACi: VPA 0.1 and 0.5 mM and SB 0.1 mM) had similar protective effects as EPO in this series of experiments (each column represents the increase in RGC survival [%] after HDACi treatment compared to individual controls [neuroprotective power=mean cell numbers treatment/mean cell numbers control*100]).

Figure 2 shows the dose-dependent effect of HDACi on RGC survival after 48 h in culture (n=4–6 culture experiments at each concentration). Compared to controls (pure media), VPA 0.1 and 0.5 mM significantly increased RGC survival to 163% (p<0.01) and 157% (p<0.05), respectively. While VPA 0.75 and 1.0 mM had no protective effect on RGCs, VPA 2.0 mM significantly decreased RGC survival to 22% (p<0.001, Figure 2A). SB 0.1 mM significantly increased RGC survival to 188% (p<0.001). A nonsignificant protective effect was seen with SB 0.5 mM. SB 1.0 mM did not affect RGC survival in comparison to control conditions. Higher concentrations of SB (≥5 mM) significantly decreased RGC survival (Figure 2B). In contrast, TSA 0.5 or 1.0 nM had no effect on RGC survival, while TSA 5 nM and 10 nM significantly decreased RGC survival to 40% (p<0.01) and 8% (p<0.001), respectively (Figure 2C). EPO was used as a positive control in this study in comparison to pure media (negative control). EPO 0.05 IU/µl significantly increased RGC survival to 164% (p<0.05), while higher concentrations were less effective but did not harm RGCs. Figure 2E summarizes the statistically significant neuroprotective effects of the HDACi compared with that of EPO. Each column represents the increase in RGC survival (%) after drug treatment compared to individual control (neuroprotective power=mean cell numbers treatment/mean cell numbers control*100). VPA 0.1 and 0.5 mM and SB 0.1 mM had similar protective effects as EPO 0.05 IU/µl in this series of experiments.

### Histone deacetylase inhibitor-induced hyperacetylation in retinal ganglion cells

**Figure 3:**
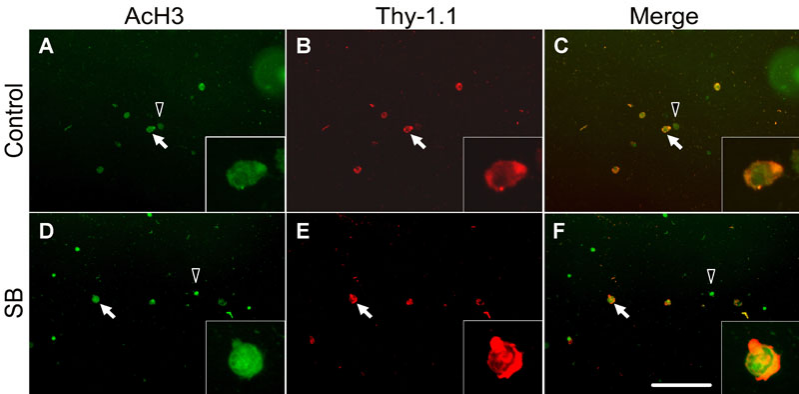
Hyperacetylation in retinal ganglion cells after histone deacetylase inhibitor treatment. Representative photographs of anti-acetylated histone proteins (AcH3; green)- and anti-Thy-1.1 (red)- labeled retinal ganglion cells (RGCs) of controls (**A**-**C**) and after sodium butyrate (SB) treatment (**D**-**F**). Thy-1.1 immunolabeling was used to specifically identify RGCs (white arrows) among other retinal cells (black arrowheads). In comparison to the control, the AcH3 immunoreactivity seems upregulated in RGCs after SB treatment. The scale bar in **F** is for all pictures and represents 100 µm. The insets show a single RGC at a higher magnification.

Immunohistochemistry was performed to determine whether HDACi treatment (SB and VPA 0.1 mM) increased the amount of AcH3 and AcH4 in RGCs. Figure 3 shows representative photographs of anti-AcH3 (green)- and anti-Thy-1.1 (red)-labeled RGCs of controls (Figure 3A-C) and after SB treatment (Figure 3D-F). Thy-1.1 immunolabeling was used to specifically identify RGCs (white arrows) among other retinal cells (black arrowheads).

**Figure 4:**
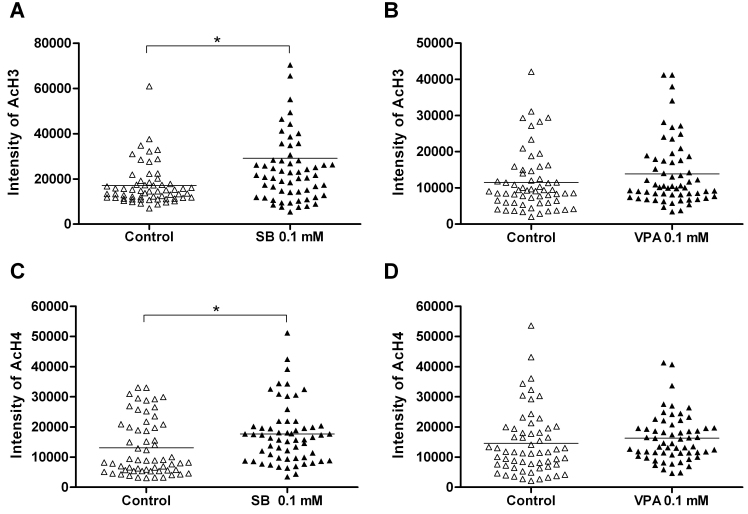
Increased acetylated histone 3 and acetylated histone 4 levels in retinal ganglion cells after histone deacetylase inhibitor treatment. **A**-**D**: Intensity of acetylated histone (AcH) 3 and AcH4 expression in retinal ganglion cells (RGCs) with or without histone deacetylase inhibitor (HDACi) treatment after 24 h in culture. **A**, **C**: In comparison to controls, sodium butyrate (SB) 0.1 mM significantly increased the amount of AcH3 (**A**, *p=0.0119) and AcH4 (**C**; *p=0.0105) in RGCs. The expression quotients were 1.7 and 1.4, respectively, indicating an apparent hyperacetylation after SB treatment. The horizontal line in **A**+**C** indicates, that the difference in hyperacetylation was statistically significant. **B**, **D**: Valproic acid (VPA) slightly increased AcH3 (**B**, p=0.1368) and AcH4 (**D**, p=0.3326) over baseline levels in controls; however, differences were not statistically significant (the expression quotients were 1.2 and 1.1, respectively).

The intensity of AcH3 and AcH4 expression was quantified in RGCs with or without HDACi treatment by determining the total number of pixels per graytone over threshold after 24 h in culture. Figure 4 shows all four groups with their corresponding negative controls. Data of 60 randomly chosen RGCs from three experiments per group are presented. In comparison to controls, SB 0.1 mM significantly increased AcH3 (17,101±1,169 versus 29,073±4,540 pixels per graytone/RGC, p=0.0119; Figure 4A) and AcH4 (13,052±1,203 versus 17,616±1,279 pixels per graytone/RGC, p=0.0105; Figure 4C) levels in RGCs, respectively. The expression quotients were 1.7 and 1.4, respectively, indicating an apparent increase of AcH3 and AcH4 in RGCs after SB treatment. Compared with the controls, VPA slightly increased AcH3 (11,544±1,061 versus 13,913±1,173 pixels per graytone/RGC, p=0.1368; Figure 4B) and AcH4 (14,598±1,371 versus 16,251±1,004 pixels per graytone/RGC, p=0.3326; Figure 4D) levels over baseline levels; however, the differences were not statistically significant (the expression quotients were 1.2 and 1.1, respectively).

## Discussion

The neuroprotective potential of HDACi has not been evaluated extensively in cultures of RGCs or animal models of optic nerve diseases. In this study we tested the effect of three HDACi on the survival of purified postnatal RGCs, which underwent spontaneous cell death over time in culture. The main findings can be summarized as follows: (1) SB and VPA enhanced RGC survival in culture to 188% and 163%, respectively, with both substances showing a maximum effect at 0.1 mM; (2) the neuroprotective effects of SB and VPA were comparable to that of EPO 0.05 IU/µl; (3) TSA 0.5–1.0 nM did not affect RGC survival but increased cell death in concentrations ≥5 nM; (4) AcH3 and AcH4 levels were significantly higher in RGCs treated with SB 0.1 mM, while VPA 0.1 mM produced only a slight effect on histone acetylation.

The role of protein acetylation has emerged as an important posttranslational modification that regulates multiple cellular functions, including chromatin remodeling and transcriptional regulation [3,7]. The upregulation of transcription via hyperacetylation can be achieved in cells either by stimulating HAT or inhibiting HDAC. HDAC have recently been recognized as potentially useful therapeutic targets in a broad range of human disorders. Pharmacological manipulations using HDACi have been beneficial in various experimental models of central nervous system diseases [15-18,32].

VPA and SB are short-chain fatty acids that readily cross the blood–brain barrier. They have been found to have low toxicity and acceptable tolerability in both human and animal studies [33,34]. We previously detected a neuroprotective effect of VPA on RGCs after ONC [25]. In the current investigation using for the first time postnatal rat RGC cultures, we found that VPA 0.1 and 0.5 mM and SB 0.1 mM significantly increased RGC survival. VPA 0.75 and 1.0 mM and SB 0.5 and 1.0 mM had no impact on RGC survival, while VPA ≥2 mM and SB ≥5 mM significantly decreased levels of viable RGCs (Figure 2A,B). These results are partly consistent with previous in vitro studies that showed a neuroprotective effect of VPA in cultures of cerebral cortical neurons (CCN) [12] or cerebellar granule cells (CGC) [9], with a maximum effect at concentrations of 0.5 mM or 1.6 mM, respectively. VPA concentrations of 0.1 to 0.4 mM and 0.75 to 1.0 mM tested in these experiments were found to be less efficient but still neuroprotective, while VPA concentrations >1.6 mM were not determined [9,12]. SB protected CGC from excitotoxicity in concentrations ranging from 0.125 to 1.0 mM [9]. The neuroprotective effect of VPA on RGCs occurred at therapeutic concentrations used for the treatment of bipolar disorder and seizures [35]. The neuroprotective potential of VPA and SB was comparable to that of EPO, an established neuroprotectant for RGCs [29-31]. Interestingly, the VPA-/SB-mediated RGC protection was not mimicked by TSA, a particularly potent HDACi. In the present investigation, TSA 0.5–1.0 nM had no impact on RGC survival but increased cell death in concentrations ≥5 nM. Previous studies have likewise revealed marked toxic effects of TSA 100 nM in neuronal cells [36] and of TSA 100, 300, and 600 nM in cultured dopaminergic neuronal cells [37]. Lower doses of TSA (1.0–10 nM) were insufficient [36]/less sufficient [37] to trigger cell death. By contrast, other authors have reported a neuroprotective effect of TSA in the following concentrations and cells: 10–30 nM in CCN cultures [12] and 25–100 nM in CGC cultures [9]. Furthermore, Schwechter et al. reported significant differentiation and neuritogenesis of RGC-5 cells after exposure to TSA 500 nM [24], and Pelzel et al. found an attenuated cell loss in the ganglion cell layer of mice after a 1 mg/kg intraperitoneal injection of TSA before ONC [23]. In summary, HDACi have mainly been tested as potential therapeutics for neurodegenerative disorders and in most cases have shown dose-dependent neuroprotective effects. However, in some cases HDACi seem to play a role in the pathogenesis of neurodegenerative diseases. This may depend on factors such as epigenetic status, cell type, and tissue specificity.

What is the basis on which SB and VPA act in this model? At present there are few data on epigenetic approaches using HDACi in the treatment of ophthalmological diseases, and the pharmacological characteristics of HDACi-induced protection of RGCs have not yet been elaborated. One of the first documentations of epigenetic changes (increased HDAC activity and decreased H4 acetylation) associated with RGC death after ONC in mice was just recently published by Pelzel et al. [23]. VPA and SB were recently found to be direct inhibitors of HDAC at clinically relevant concentrations [8,38]. The VPA-induced neuroprotection in neuronal cultures was therefore associated with a robust increase in AcH3 levels [9,12,39]. In the present investigation, SB and VPA but not TSA enhanced RGC survival, although AcH3 and AcH4 levels were only significantly higher in RGCs treated with SB. Thus, histone acetylation might play a subordinate role next to other potential neuroprotective pathways acting in this model.

In our recent study, the VPA-mediated neuroprotection after ONC was accompanied by decreased caspase-3 activity, cyclic adenosine monophosphate (cAMP) response element binding protein (CREB) induction, and phosphorylated extracellular signal-regulated kinase (pERK) 1/2 activation but not by altered histone acetylation, as shown in western blots [25]. In addition to histone acetylation, HDACi have been reported to exert neuroprotective effects by enhancing the acetylation of cytoprotective transcription factors and by inhibiting some forms of apoptosis [40]. VPA treatment, for example, dramatically enhanced the acetylation of nuclear factor-kappa-light-chain-enhancer of activated B-cells (NF-kB) in neuronal cells kept under hypoxia for 6 h [39]. The anti-apoptotic activity of NF-kB was mediated by inducing the transcription of several anti-apoptotic genes [39]. Furthermore, VPA has been shown to protect CCN against low K^+^-induced apoptosis by acting on the phosphatidylinositol 3-kinase/protein kinase B pathway [41] and to activate the ERK pathway, thereby inducing ERK pathway-mediated neurotrophic actions, such as neurite growth, regeneration, and neurogenesis [13,25]. The more recent data on VPA neuroprotective mechanisms in models of neurodegenerative diseases were summarized by Monti and colleagues [42] who demonstrated that VPA affects several fundamental cellular processes by targeting multiple molecular mechanisms. Moreover, beside direct effects of HDACi on neurons, recent reports have shown their immunomodulatory action on microglia [20,43]. VPA, TSA, and SB have been shown to induce an apoptosis-related decrease of cultured rat microglia, which occurs with a reduced neuroinflammatory response in lipopolysaccharide (LPS)-treated neuron-glia cultures, thereby acting neuroprotectively [44]. Additional studies are necessary to identify HDACi-targeted genes and pathways involved in RGC protection.

Our study has some limitations. First, it raises no claim to completeness regarding the analysis of acetylation-associated processes. Therefore, the hypothesis of RGC protection through hyperacetylation should not be rejected. We only analyzed the acetylation levels of histone proteins 3 and 4, which were described as the most commonly involved histones that are altered in models of neurodegeneration. To further characterize the role of modulated histone acetylation in models of optic nerve injury, e.g., other histone proteins and, the activity of HAT and HDAC should be examined as well as the acetylation of cytoprotective transcription factors. However, due to limited amounts of sample material available for further molecular investigation using purified postnatal RGC cultures, other in vivo models should be used to identify HDACi-associated pathways and mechanism involved in RGC protection. Another point of criticism is that VPA and SB had only little therapeutic range, with higher doses causing RGC degeneration. Instead of being neuroprotective, VPA can even exacerbate neuronal death under some conditions [45]. A possible explanation with regard to acetylation is that the modes of action of HDACi are highly nonspecific and nontargeted; they may reverse the deacetylation-mediated blockade of undesirable nonspecific promoters, leading to cytotoxicity. These findings suggest a need for caution in studies that attempt to examine the molecular mechanisms of HDACi-mediated neuroprotection. Another potential shortcoming of this investigation is its open-label design due to logistical reasons. We tried to circumvent this problem by carrying along two control groups, a negative cohort with media alone and a second cohort with an effective treatment (EPO). Furthermore, the morphologic characteristics of viable RGCs were clearly defined.

Taken together, HDACi VPA and SB are promising candidates to counteract neuronal loss in the brain and possibly in ophthalmological diseases. The present study is the first to detect HDACi-mediated hyperacetylation in purified rat RGCs, although significantly elevated AcH3 and AcH4 levels were only present in RGCs after SB treatment. Further studies are needed to identify HDACi-targeted genes and pathways involved in RGC protection and to assess in detail the biologic potency, safety, and pharmacotoxicity of these drugs with regard to the retina.
